# Self-rated health of Brazilian women of childbearing age: a cross-sectional study

**DOI:** 10.1590/1980-220X-REEUSP-2023-0127en

**Published:** 2023-10-30

**Authors:** Luiza Oliveira Santos, Thayane Fraga de Paula, Maria José Silva Souza, Bruna Nicole Soares dos Santos, Erica Dumont Pena, Mariana Santos Felisbino-Mendes

**Affiliations:** 1 Universidade Federal da Paraíba, Centro de Ciências da Saúde, Núcleo de Estudos em Saúde Coletiva, João Pessoa, PB, Brazil.; 2 Universidade Federal de Minas Gerais, Escola de Enfermagem, Departamento de Enfermagem Materno Infantil e Saúde Pública, Belo Horizonte, MG, Brazil.

**Keywords:** Self-Testing, Noncommunicable Diseases, Risk Factors, Women’s Health, Autoevaluación, Enfermedades no Transmisibles, Factores de Riesgo, Salud de la Mujer, Autoavaliação, Doenças não Transmissíveis, Fatores de Risco, Saúde da Mulher

## Abstract

**Objective:**

To investigate sociodemographic factors, non-communicable diseases and conditions, and behavioral risk factors associated with negative self-rated health among Brazilian women of childbearing age.

**Method:**

Cross-sectional study with 26,071 Brazilian women of reproductive age. Estimated prevalence of self-rated health according to sociodemographic characteristics, non-communicable diseases and conditions, and behavioral risk factors. Poisson regression was used to estimate adjusted and unadjusted prevalence ratios.

**Results:**

Occurrence of two or more of the diseases and conditions presented a prevalence of negative self-rated health almost three times higher than none. There was a positive association between negative self-rated health and older age groups, lower education, black or brown skin color/race, living in the north and northeast regions, physical inactivity, being a smoker, and presence of one or more of the diseases and conditions.

**Conclusion:**

There are differences in self-rated health, reflecting social inequalities.

## RESUMO

**Objetivo:**

Investigar fatores sociodemográficos, doenças e agravos não transmissíveis e fatores de risco comportamentais associados à autoavaliação de saúde negativa das mulheres brasileiras em idade reprodutiva.

**Método:**

Estudo transversal com 26.071 mulheres brasileiras em idade reprodutiva. Estimadas prevalências da autoavaliação de saúde segundo características sociodemográficas, doenças e agravos não transmissíveis e fatores de risco comportamentais. Utilizou-se regressão de Poisson para estimar as razões de prevalência ajustadas e não ajustadas.

**Resultados:**

Ocorrência de duas ou mais das doenças e agravos apresentou prevalência de autoavaliação de saúde negativa quase três vezes maior do que nenhuma. Houve associação positiva entre autoavaliação de saúde negativa e maiores faixas etárias, menor escolaridade, cor da pele/raça preta ou parda, viver nas regiões norte e nordeste, inatividade física, ser fumante e presença de uma ou mais das doenças e agravos.

**Conclusão:**

Existem diferenças na autoavaliação de saúde, refletindo em iniquidades sociais.

## RESUMEN

**Objetivo:**

Investigar factores sociodemográficos, enfermedades y condiciones no transmisibles y factores de riesgo conductuales asociados con la autoevaluación negativa de la salud entre mujeres brasileñas en edad fértil.

**Método:**

Estudio transversal con 26.071 mujeres brasileñas en edad reproductiva. Prevalencia estimada de salud autovalorada según características sociodemográficas, enfermedades y trastornos no transmisibles y factores de riesgo conductuales. Se utilizó la regresión de Poisson para estimar las tasas de prevalencia ajustadas y no ajustadas.

**Resultados:**

La aparición de dos o más de las enfermedades y agravios mostró una prevalencia de autoevaluación negativa de la salud casi tres veces mayor que ninguna. Hubo una asociación positiva entre la autoevaluación negativa de la salud y los grupos de mayor edad, menor educación, color de piel/raza negra o morena, vivir en las regiones norte y noreste, inactividad física, ser fumador y la presencia de una o más de las enfermedades y condiciones.

**Conclusión:**

Existen diferencias en la autoevaluación de la salud, lo que refleja desigualdades sociales.

## INTRODUCTION

Women’s health care has historically developed under a reductionist perspective focused on motherhood and reproduction, affecting mainly women of childbearing age^(1)^. This paradigm, which reproduces aspects of gender inequality, prevents women from being fully assisted, reducing opportunities to achieve better levels of health^(1)^. Most publications on policies aimed at women’s health address sexual and reproductive issues and, although non-communicable diseases and conditions (NCDs) are the main causes of death and disability for women^(2)^, publications related to NCDs and their risk factors are rare.

In this context, analyzing indicators produced by population surveys would allow identifying women’s health needs and their determinants, contributing to a comprehensive health agenda. As part of these indicators, there is the self-rated health (SRH), a measure that summarizes the general health status^(3)^. Commonly measured in population surveys as a subjective measure^(4)^, it is believed that this multidimensional construct^(3,5,6)^ is able to reflect not only biological factors^(6)^, but also psychological^(7)^ and social^(5)^ ones. Therefore, SRH points out the impacts that health problems and psychosocial conditions cause on people’s lives and can indicate the notion of having a healthy life or not^(8)^.

The conditions influencing SRH described in the national and international literature are sociodemographic characteristics^(3,9–11)^, presence of diseases and conditions^(6,8,11,12)^, and health behaviors^(4,5,11)^. Morever, more recent publications have mostly investigated SRH in the general population^(3,6,9,11)^. Few studies were found that specifically addressed women^(10,11,13,14)^, with women of childbearing age included in only two of these studies^(13,14)^.

There is evidence of the association of negative SRH with low educational level^(3–6)^, smoking^(4,5)^, physical inactivity^(5)^, presence of diseases^(4,6,12)^, and simultaneous occurrence of morbidities^(12)^. With regard strictly to women, the most evident associations were those with the presence of diseases^(11)^ and low education^(10,11)^. For women of childbearing age, an Iranian study showed an association with low education and low income^(13)^.

Besides the scarcity of studies on SRH specifically addressing women of childbearing age, it is worth considering the misconception that NCDs are male disorders^(15)^, the different perceptions about one’s own health within different groups^(3)^, and the SRH as a reliable measure of health status^(12)^.

Thus, the present study aimed to investigate the sociodemographic factors, non-communicable diseases and conditions, and their behavioral risk factors associated with the negative self-rated health of these women in Brazil.

## METHOD

### Design of Study

This is a cross-sectional, population-based study. Data from the *Pesquisa Nacional de Saúde* (*PNS*), or National Health Survey, developed in 2019 and which aimed to generate information on the Brazilians’ health status and lifestyle, as well as to produce data on the operation of health services in the country^(16)^ were analyzed.

### Population, Local, and Selection Criteria

The target population of the PNS consisted of individuals residing in the national territory in permanent private households, that is, places built with the exclusive purpose of housing^(16)^. To compose the representative sample of the Brazilian population, three-stage cluster sampling was used^(16)^. At the end of the stages, 94,114 home interviews and 90,846 individual interviews were carried out^(16)^. For this study, the subpopulation of women of reproductive age aged 18 years or older was selected, including the 26,071 women aged 18 to 49 who responded the survey.

As an outcome, the SRH was considered and, based on the selected resident’s questionnaire, the question “In general, how do you assess your health?” was used. Based on the responses, this variable was categorized as very good/good, fair, and poor/very poor.

As NCDs, systemic arterial hypertension, diabetes mellitus, and asthma were considered, with self-reported yes or no answers. Behavioral risk factors (BRF) were analyzed: alcohol consumption (yes or no), tobacco use (non-smoker, ex-smoker, or smoker), intake of fruits and vegetables (enough - five or more servings per day – or insufficient – less than five servings a day)^(17)^ and physical activity (active – at least 150 minutes of light/moderate activity per week or 75 minutes of vigorous activity per week – or inactive, less than 150 minutes of light/moderate activity per week or less than 75 minutes of vigorous activity per week)^(18)^.

In addition, aiming to assess whether the simultaneous occurrence of diseases and conditions is associated with SRH, the NCD responses were added, building a simultaneity score, creating a counting variable that includes none, one, and two or more NCD. The same analysis was carried out for the BRFs.

The following sociodemographic characteristics were considered: age group (18–29, 30–39, 40–49 years old); level of education (0–9, 10–12, 13 or more years of study), skin color/race (white, brown/black, yellow/indigenous); region of residence (southeast, south, midwest, northeast, north), and marital status (with and without partner).

To investigate the association between the independent variables and the SRH, it was grouped into two categories: positive (responses “very good” and “good”) and negative (responses “fair”, “poor”, “very poor”).

### Data Analysis

Prevalence was estimated with 95% confidence intervals (95%CI) for SRH, sociodemographic characteristics, NCDs, and BRFs. Subsequently, SRH prevalence was estimated according to sociodemographic characteristics, the presence of NCDs and BRFs. To verify statistical differences between groups, Pearson’s chi-square test was used with a significance level of 5%.

Poisson regression^(19)^ of each independent variable with the SRH was performed, which obtained unadjusted prevalence ratios (PR) and 95% CI. Variables with p ≤ 0.20 were included in the multivariate model to obtain adjusted prevalence ratios (aPR). The method backwards was used, with those with a significance level of p ≤ 0.05 remaining. For the final model, the NCD counting variable was chosen to analyze the simultaneous occurrence of diseases and conditions. To perform these analyses, the statistics software Stata 14.0, module survey was used, to obtain population estimates, considering the complex sampling design of the research (stratum, cluster, and individual weight).

### Ethical Aspects

The data used in this study are in the public domain, aggregated, do not involve the identification of subjects and comply with the ethical recommendations of Resolution No. 466, of December 12, 2012, of the National Health Council.

## RESULTS

A total of 26,071 Brazilian women of childbearing age were found and, based on population estimates, it was observed that the majority were between 18 and 29 years old (34.9%) and 30 and 39 years old (34%), 10 to 12 years of study (46.3%) and 13 or more years of study (26.9%), were black or brown (58.3%), had no partner (62.4%), lived in the southeast (42.1%) and northeast (27.3%) regions (data not shown in tables) and assessed their health as very good/good (71.5%) (Table 1).

**Table 1:** Prevalence of self-rated health (SRH), behavioral risk factors (BRF), and non-communicable diseases and conditions (NCD) among Brazilian women of childbearing age – Brazil, 2019.

Variables	%* (95%CI)
***SRH***	
Very good/Good	71.5 (70.6–72.5)
Fair	24.6 (23.7–25.6)
Bad/Very bad	3.9 (3.5–4.3)
***BRF***	
Physical inactivity	71.2 (70.2–72.2)
Insufficient consumption of FV	91.6 (91.0–92.2)
Alcohol consumption	38.7 (37.5–39.9)
Ex-smokers	21.1 (20.2–22.0)
Smokers	8.4 (7.9–9.0)
***BRF count***	
None	2.1 (1.8–2.4)
One	15.3 (14.6–16.2)
Two or more	82.6 (81.7–83.4)
***NCD***	
Systemic arterial hypertension	13.9 (13.1–14.8)
Asthma	6.4 (5.9–7.0)
Diabetes mellitus	3.4 (3.0–3.8)
***NCD count***	
None	78.8 (77.8–79.7)
One	18.4 (17.6–19.2)
Two or more	2.8 (2.4–3.2)

*population estimates; 95% CI: 95% confidence interval; SRH: self-rated health; BRF: behavioral risk factors; FV: fruit and vegetables; NCD: non-communicable diseases and conditions.

Most women were physically inactive and consumed an insufficient portion of FVs. For every ten women, eight or more had two or more BRFs. Regarding NCDs, 13.9% had systemic arterial hypertension, 3.4% diabetes mellitus, and 6.4% asthma (Table 1).

It was observed that women aged 40-49 years, with low education (0–9 years), black or brown, and from the North and Northeast regions had worse self-rated health (Table 2). Furthermore, having a partner or not did not influence SRH (Table 2).

**Table 2:** Prevalence and unadjusted prevalence ratio (PR) of self-rated health (SRH) according to sociodemographic characteristics of Brazilian women of reproductive age – Brazil, 2019.

SRH					
Sociodemographic characteristics	Positive	Negative	p-value*	PR (CI95%)	p-value
	% (95%CI)	% (95%CI)			
***Age (years)***					
18–29	77.6 (75.8–79.3)	22.4 (20.7–24.2)	<0.0001	Ref.	Ref.
30–39	72.8 (71.2–74.3)	27.2 (25.7–28.8)		1.21 (1.10–1.34)	<0.0001
40–49	63.4 (61.5–65.2)	36.6 (34.8–38.5)		1.63 (1.48–1.80)	<0.0001
***Education (years)***					
0–9	57.3 (55.2–59.5)	42.7 (40.5–44.9)	<0.0001	2.82 (2.53–3.13)	<0.0001
10–12	72.0 (70.5–73.5)	28.0 (26.5–29.5)		1.85 (1.67–2.05)	<0.0001
≥13	84.9 (83.5–86.2)	15.1 (13.8–16.6)		Ref.	Ref.
***Skin color/race***					
White	77.7 (76.1–79.1)	22.3 (20.9–23.9)	<0.0001	Ref.	Ref.
Black/Brown	67.3 (66.0–68.6)	32.7 (31.4–34.0)		1.46 (1.35–1.58)	<0.0001
Yellow/Indigenous	69.1 (60.0–77.0)	30.9 (23.0–40.0)		1.38 (1.04–1.83)	0.024
***Marital status***					
With a partner	71.7 (70.2–73.2)	28.3 (26.8–29.8)	0.8099	Ref.	Ref.
Without a partner	71.4 (70.2–72.7)	28.6 (27.3–29.8)		1.01 (0.94–1.08)	0.810
***Region of residence***					
Southeast	75.3 (73.5–77.0)	24.7 (23.0–26.6)	<0.0001	Ref.	Ref.
South	79.8 (77.9–81.6)	20.2 (18.4–22.1)		0.82 (0.72–0.92)	0.001
Midwest	74.1 (71.9–76.2)	25.9 (23.8–28.1)		1.05 (0.94–1.17)	0.420
North East	62.5 (60.8–64.2)	37.5 (35.8–39.2)		1.52 (1.39–1.65)	<0.0001
North	66.2 (64.2–68.1)	33.8 (31.9–35.8)		1.37 (1.24–1.50)	<0.0001

*Pearson’s chi-square test; PR: unadjusted prevalence ratio; 95% CI: 95% confidence interval.

In the presence of BRF, the prevalence of SRH among the women studied remained predominantly positive, but physical inactivity, insufficient intake of FV, and tobacco use were associated with negative SRH (Table 3). In addition, the occurrence of NCD reflected higher prevalence of negative SRH and, the higher the number of NCD, the worse the women assessed their health. It was also observed that diabetes was the condition that most affected SRH, with the highest prevalence of negative responses (Table 3).

**Table 3:** Prevalence and prevalence ratio (PR) of unadjusted self-rated health according to the behavioral risk factors (BRF) and non-communicable diseases and conditions (NCD) among Brazilian women of childbearing age – Brazil, 2019.

SRH					
BRF and NCD	Positive	Negative	p-value*	PR	p-value
	% (95%CI)	% (95%CI)		% (95%CI)	
**Physical activity**					
Active	79.0 (77.4–80.5)	21.0 (19.5–22.6)	<0.0001	Ref.	Ref.
Inactive	68.5 (67.3–69.7)	31.5 (30.3–32.7)		1.50 (1.38–1.62)	<0.0001
***FV Intake***					
Enough	76.6 (73.4–79.5)	23.4 (20.5–26.6)	0,0016	Ref.	Ref.
Insufficient	71.1 (70.0–72.1)	28.9 (27.9–30.0)		1.24 (1.08–1.42)	0.002
***Alcohol consumption***					
No	69.2 (68.0–70.5)	30.8 (29.5–32.0)	<0.0001	Ref.	Ref.
Yes	75.2 (73.7–76.7)	24.8 (23.4–26.4)		0.81 (0.75–0.87)	<0.0001
***Tobacco use***					
Non-smoker	73.5 (72.3–74.6)	26.5 (25.4–27.7)	<0.0001	Ref.	Ref.
Ex-smoker	68.2 (66.1–70.2)	31.8 (29.8–33.9)		1.20 (1.11–1.30)	<0.0001
Smoker	63.5 (60.0–66.9)	36.5 (33.1–40.0)		1.38 (1.24–1.53)	<0.0001
***BRF count***					
None	83.1 (77.8–87.3)	16.9 (12.7–22.2)	<0.0001	Ref.	Ref.
One	75.5 (73.2–77.7)	24.5 (22.3–26.8)		1.45 (1.08–1.93)	0,012
Two or more	70.5 (69.4–71.5)	29.5 (28.5–30.6)		1.74 (1.32–2.30)	<0.0001
***Arterial hypertension***					
No	75.1 (74.0–76.1)	24.9 (23.9–26.0)	<0.0001	Ref.	Ref.
Yes	49.1 (46.5–51.8)	50.9 (48.2–53.5)		2.03 (1.90–2.18)	<0.0001
***Asthma***					
No	72.2 (71.2–73.2)	27.8 (26.8–28.8)	<0.0001	Ref.	Ref.
Yes	61.5 (56.9–65.8)	38.5 (34.2–43.0)		1.39 (1.23–1.57)	<0.0001
***Diabetes mellitus***					
No	72.7 (71.6–73.7)	27.3 (26.3–28.4)	<0.0001	Ref.	Ref.
Yes	39.5 (33.7–45.7)	60.5 (54.3–66.3)		2.21 (1.98–2.46)	<0.0001
***NCD count***					
None	76.4 (75.2–77.5)	23.6 (22.5–24.8)	<0.0001	Ref.	Ref.
One	56.4 (54.0–58.7)	43.6 (41.2–46.0)		1.85 (1.72–1.99)	<0.0001
Two or more	35.3 (28.9–42.4)	64.7 (57.6–71.2)		2.74 (2.43–3.08)	<0.0001

*Pearson’s chi-square test; BRF: behavioral risk factors; NCD: non-communicable diseases and conditions; PR: unadjusted prevalence ratio; 95% CI: 95% confidence interval; FV: fruit and vegetables.

In the final model, after adjustments, the maintenance of the positive association between negative SRH and higher age ranges (40–49 years), lower education (0–9 years and 10–12 years, with dose-response relationship), black or brown skin color/race and North and Northeast regions (Table 4) was observed. Analyzing the BRCs, physical inactivity and being a smoker were also positively associated with a fair/poor/very poor assessment (Table 4).

On the other hand, women who consume alcohol assess their health negatively in a lower prevalence (Table 4). In addition, those women with two or more NCD had a prevalence of negative self-rated health twice as high as those with no NCDs (Table 4), also with a dose-response relationship.

**Table 4:** Adjusted prevalence ratios (aPR) of negative self-rated health according to the sociodemographic characteristics, behavioral risk factors (BRF), and non-communicable diseases and conditions (NCD) among Brazilian women of childbearing age – Brazil, 2019.

Sociodemographic characteristics, BRF and NCD	aRP (95% CI)	p-value
***Age (years)***		
18–29	Ref.	Ref.
30–39	1.14 (1.03–1.25)	0.008
40–49	1.34 (1.22–1.48)	<0.0001
**Education (years)**		
0–9	2.04 (1.82–2.28)	<0.0001
10–12	1.66 (1.50–1.85)	<0.0001
≥13	Ref.	Ref.
***Skin color/race***		
White	Ref.	Ref.
Black/Brown	1.14 (1.05–1.24)	0.002
Yellow/Indigenous	1.28 (0.96–1.69)	0.09
***Region of residence***		
Southeast	Ref.	Ref.
South	0.85 (0.76–0.96)	0.007
Midwest	1.07 (0.96–1.19)	0.219
North East	1.40 (1.29–1.53)	<0.0001
North	1.31 (1.19–1.45)	<0.0001
***Behavioral risk factors***		
Physical inactivity	1.28 (1.18–1.39)	<0.0001
Alcohol consumption	0.89 (0.82–0.97)	0.006
Ex-smoker	1.16 (1.06–1.26)	<0.0001
Smoker	1.23 (1.09–1.38)	<0.0001
***NCD count***		
None	Ref.	Ref.
One	1.65 (1.53–1.77)	<0.0001
Two or more	2.18 (1.94–2.45)	<0.0001

*95%CI: 95% confidence interval; BRF: behavioral risk factors; NCD: non-communicable diseases and conditions; aRP: adjusted prevalence ratio.

## DISCUSSION

Brazilian women of childbearing age aged between 40 and 49 years, with low level of education, black or brown skin color, living in the North and Northeast regions had worse self-rated health and, the lower the education, the higher the prevalence of negative SRH.

Positive SRH does not necessarily imply better health or more realistic knowledge about one’s own body. In this study, the presence of BRF in health impacted the SRH of the women studied, being negative and indicating that Brazilian women show understanding about their health status.

A previous study showed that women of childbearing age with higher education and income also had a better sense of their own health^(13,14)^, just as a study carried out with climacteric women showed that negative SRH was significantly associated with low level of education^(11)^, corroborating the findings of the present study.

It is believed that low education, black and brown skin color and residing in marginalized regions imply less possibilities of social participation and maintenance of healthy lifestyle habits^(6,11,13)^, conditions that are not under the control of individuals and that repress the enjoyment of full autonomy^(20)^. Skin color, which in Brazil is intrinsically associated with the class dimension, is also described in a North American study in which black people assessed their health in a significantly worse manner when compared to white people^(9)^. It is clear, then, that social and racial factors can affect the health-disease processes and imply a negative self-assessment of one’s own health.

Although the negative SRH may represent an overload for the lives of poor, black/brown women with less education, the self-recognition of health weaknesses, in this case, also indicates a conscious perspective on their reality. The idea of consciousness triggered here is related to the Freirean perspective that people, by assuming themselves as historical subjects, perceive reality and create conditions to rebuild it^(21)^. Thus, the awareness of these women, reflected in their negative SRH, would still oppose the discourse – present in common sense – that less educated and poor people would have less awareness, which refers to the idea of awareness related to the ability to apprehend school content.

In this study, it was also observed that the greater the number of diseases and conditions, the greater the prevalence of negative SRH, and being physically inactive and having diabetes were the conditions that most affected SRH, revealing that different diseases are perceived in different ways. This can be explained by the legitimacy given to each process of illness, since symptomatic conditions, such as diabetes, are considered more harmful to health than those that are said to be silent, such as hypertension^(22)^.

Damage to health is better noted when there is one or more NCD, with a dose-response relationship. This does not occur in relation to BRF, thus referring to the concept of health as the absence of disease. This perception is reaffirmed by the current practice of Brazilian health care which, despite recommending the prioritization of health promotion and disease prevention actions, still has actions guided and with greater emphasis on healing^(6)^, although it is well established that these risk factors are also harmful to health and increase the likelihood of NCD development^(23)^.

These findings reinforce the invisibility of women in the context of NCD and their BRF, already highlighted in the literature^(2,15)^. Despite being the main users of health services in Brazil, they are not fully assisted, reflecting the invisibility of BRF for women themselves, especially to the younger and asymptomatic ones. Assistance to these women is usually centered on specific protocols, such as prenatal and postpartum care, reproductive planning and prevention of breast and cervical cancer, leading to fragmentation of care.

Regarding BRFs, in this study being a smoker was associated with negative SRH, which was not observed in relation to alcohol consumption. Since both are licit drugs, this difference may be mainly related to the social acceptance of alcohol and measures to prevent tobacco consumption. Tobacco consumption undergoes intense regulatory measures, and between 2006 and 2013, an improvement in smoking indicators was observed in both sexes, different levels of education, age, and regions^(23,24)^. On the other hand, in the same period, there was an increase of 3.8% per year in alcohol abuse among women aged 30 to 39 years^(25)^. Alcohol consumption is culturally associated with moments of relaxation, leisure, and well-being, and its consumption is often encouraged, hindering the comprehension of the damage harmful to the body. Therefore, it is believed that alcohol consumption does not protect against negative SRH, but that this finding is related to positive social recognition of its use.

Also regarding BRFs, insufficient intake of FVs was the most prevalent factor among Brazilian women of childbearing age, as well as being associated with SRH, that is, food influences the perception one has about one’s own health.

Among the limitations of the study, it should be noted that, in the PNS, the SRH is objectively measured, and does not work with its narratives. Another possible limitation is the self-reported variables that may eventually underestimate prevalence, a conservative bias.

It is considered that these findings contribute to recognizing inequalities in the health of Brazilian women of childbearing age, since it is a database with national representativeness, and may constitute a reference for future studies^(26)^ and guide policies in the field of women’s health. In addition, it allows identifying women’s health needs in a multidimensional and broader perspective within the scope of NCD.

## CONCLUSION

There are differences in the SRH that reflect a more frequent negative assessment by black or brown, older women, with low level of education, and residents of the North and Northeast regions, regardless of the presence of NCD and its risk factors. However, this assessment, in addition to reflecting social inequalities, may indicate women’s awareness of their historical trajectories, permeated by inequalities and the possibility of change, reflected in critical awareness. In addition, women recognize more damage to their health in the presence of NCDs and risk factors such as smoking and physical inactivity. These findings point to the need to rethink current public policies and programs with the appreciation of actions aimed at equity and integrality to the health of Brazilian women of childbearing age.
